# Associations between the triglyceride-glucose index and cardiovascular disease in over 150,000 cancer survivors: a population-based cohort study

**DOI:** 10.1186/s12933-022-01490-z

**Published:** 2022-04-16

**Authors:** Mi-Hyang Jung, Sang-Wook Yi, Sang Joon An, Jee-Jeon Yi, Sang-Hyun Ihm, Seongwoo Han, Kyu-Hyung Ryu, Hae Ok Jung, Ho-Joong Youn

**Affiliations:** 1 Division of Cardiology, Department of Internal Medicine, Seoul St. Mary’s Hospital, College of Medicine, The Catholic University of Korea, Seoul, 06591 Republic of Korea; 2 Catholic Research Institute for Intractable Cardiovascular Disease College of Medicine , The Catholic University of Korea , 06591 Seoul, Republic of Korea; 3 Department of Preventive Medicine and Public Health, College of Medicine, Catholic Kwandong University, Gangwon-do 25601 Gangneung, Republic of Korea; 4 Department of Neurology , International St. Mary’s Hospital Catholic Kwandong University College of Medicine , 22711 Incheon, Republic of Korea; 5 Institute for Occupational and Environmental Health, Catholic Kwandong University, Gangneung, 25601 Republic of Korea; 6 Division of Cardiology, Department of Internal Medicine, Bucheon St. Mary’s Hospital, College of Medicine, The Catholic University of Korea, Bucheon-si, 14647 Republic of Korea; 7 Cardiovascular Center, Dongtan Sacred Heart Hospital Hallym University College of Medicine, 18450 Hwaseong-si, Republic of Korea

**Keywords:** Cancer, Cardio-oncology, Survivorship, Cardiovascular diseases, Prevention

## Abstract

**Background:**

The prevention of subsequent cardiovascular disease (CVD) is an essential part of cancer survivorship care. We conducted the present study to investigate the association between the TyG index (a surrogate marker of insulin resistance) and the risk of cardiovascular disease (CVD) events in cancer survivors.

**Methods:**

Adult cancer patients, who underwent routine health examinations during 2009–2010 and were survived for more than 5 years as of January 1, 2011, were followed for hospitalization of CVD (either ischemic heart disease, stroke, or heart failure) until December 2020. Cox model was used to calculate hazard ratios associated with baseline TyG index (log_e_ [fasting triglyceride (mg) × fasting glucose (mg)/2]) for the CVD hospitalization.

**Results:**

A total of 155,167 cancer survivors (mean age 59.9 ± 12.0 years, female 59.1%) were included in this study. A graded positive association was observed between TyG and CVD hospitalization. An 8% elevated risk for CVD hospitalization was observed for a TyG index of 8-8.4 (aHR 1.08 [95% CI 1.01–1.14]); 10% elevated risk for a TyG index of 8.5–8.9 (aHR 1.10 [95% CI 1.03–1.17]); 23% elevated risk for a TyG index of 9.0-9.4 (aHR 1.23 [95% CI 1.15–1.31]); 34% elevated risk for a TyG index of 9.5–9.9 (aHR 1.34 [95% CI 1.23–1.47]); and 55% elevated risk for a TyG index **≥** 10 compared to the reference group (TyG index < 8). Per 1-unit increase in the TyG index, a 16% increase in CVD hospitalization and a 45% increase in acute myocardial infarction hospitalization were demonstrated. Graded positive associations were evident for atherosclerotic CVD subtypes, such as ischemic heart disease, acute myocardial infarction, and ischemic stroke, but not for hemorrhagic stroke or heart failure.

**Conclusions:**

The TyG index may serve as a simple surrogate marker for the risk stratification of future CVD events, particularly atherosclerotic subtypes, in cancer survivors.

**Supplementary Information:**

The online version contains supplementary material available at 10.1186/s12933-022-01490-z.

## Background

Increasingly many cancer patients are surviving due to early diagnoses and advances in cancer therapy. Approximately 14.5 million cancer survivors are living in the United States as of 2014, and this number is expected to grow to 19 million by 2024 [1]. Cardiovascular disease (CVD) is the second most frequent cause of death in cancer survivors, following death from cancer [2, 3]. In some cancers, a gradual increase in CVD mortality during follow-up has been demonstrated, along with stable or decreasing cancer mortality [3, 4]. Thus, the prevention of CVD is an important part of cancer survivorship [3–6]. Despite its relevance, this issue has not received adequate attention, most likely because there have been more concerns related to cancer recurrence, chemotherapy-induced cardiomyopathy, and the loss of patients during the transition period from tertiary care to primary care.

The triglyceride-glucose (TyG) index has been introduced as a surrogate marker of insulin resistance that can be easily calculated using fasting glucose and triglyceride levels without sampling serum insulin [7, 8]. Previous studies have shown that the TyG index was associated with metabolic disease, subclinical atherosclerosis, and CVD [9–14]. However, the association between the TyG index and CVD in cancer survivors, who are at higher risk for subsequent CVD [2, 15, 16], is still unknown. Understanding the association between the TyG index and CVD will help us identify individuals with high CVD risk among cancer survivors, who need adequate attention and a proactive preventive strategy for subsequent CVD.

Therefore, we conducted the present study to identify the association between the TyG index and future CVD development in a large population-based cohort of cancer patients who survived for more than 5 years beyond the diagnosis of cancer and who were initially free of CVD.

## Methods

### Study database and study population

We used the Korean general health screening database linked with the Korean National Health Insurance Service (NHIS) database. The NHIS provides mandatory health insurance for 97% of the Korean population and nationwide biennial health screenings. The NHIS database contains data regarding demographic characteristics, diagnosis by International Classification of Disease (ICD) codes, prescriptions, death, and health screening examination information (anthropometric measurements, blood pressure, laboratory tests, and health questionnaires) [17].

We identified 18- to 99-year-old individuals who were diagnosed with cancer during 2002–2005 and received health screening examinations during 2009–2010. The follow-up started on January 1, 2011 (the index date). An individual was defined as having cancer if he or she was hospitalized with an ICD code for cancer (C00-97) or received outpatient or inpatient care with a critical condition code for cancer (V193, V194). We excluded those with missing values for the health examinations, prescription of triglyceride-lowering agents (fibrates, niacin, or omega-3), known CVD or diabetes before the health examination, and death before the index date. The purpose of excluding individuals with known diabetes (potentially on glucose-lowering agents) or those on triglyceride-lowering agents was to examine the association in persons not receiving treatment for lowering fasting glucose or triglyceride levels. A total of 155,167 cancer survivors constituted the final study population. A detailed flowchart of the study population is provided in Additional file 1: Fig. S1. The current study was approved by the institutional review board, and the requirement for informed consent was waived, as anonymized data were provided by the NHIS under the strict confidentiality protocol.

### Data collection

Venous samples, which included fasting glucose and lipid profiles, were obtained after overnight fasting [18]. Blood pressure was measured after **≥** 5 min of rest at least twice by trained staff members. Body mass index (BMI) was calculated as weight divided by the square of height (kg/m^2^). Data on alcohol consumption, smoking status, physical activity, and known CVD were collected via self-reported questionnaire [19]. Health examinations and data collection followed a standard protocol documented by the Ministry of Health and Welfare. More detailed information regarding the NHIS health examinations can be found elsewhere [17]. Household income status was stratified based on the quartile of all NHIS beneficiaries.

### TyG index calculation and the study outcomes

The TyG index can be calculated as follows: log_e_ [fasting triglyceride (mg) $\times$ fasting glucose (mg)/2] (7,8). The study population was categorized into 6 groups by the level of TyG: < 8 (reference), 8–8.4, 8.5–8.9, 9.0–9.4, 9.5–9.9, and **≥** 10. A primary cardiovascular event was defined as hospitalization due to major CVD (either ischemic heart disease [IHD, I20–I25], stroke [I60–I69], or heart failure [I11, I13, I255, I42, I50]). Additionally, we explored the association between the TyG index and event subtypes (hospitalization due to acute myocardial infarction [AMI, I21], ischemic stroke [I63], and hemorrhagic stroke [I60–I62]). The validity and accuracy of the codes for CVD from the NHIS data have been tested through the formation of the Event Validation Committee, and 93% of AMI events between 2008 and 2011 were validated [20]. Another study reported that > 90% of ischemic stroke and intracerebral hemorrhage codes were validated by medical records [21]. The study population was followed until the first cardiovascular event or the end of the study (December 2020).

### Statistical analysis

The baseline characteristics of study population were described as the mean ± standard deviation for continuous variables or number with percentage for categorical variables. Cox proportional hazards models were applied to evaluate the associations of the TyG index with cardiovascular events and to calculate hazard ratios (HRs), after adjustment for potential covariates, such as age, sex, household income, behavioral factors (alcohol consumption, smoking status, and physical activity), and cardiometabolic factors (systolic blood pressure, body mass index, lipid-lowering medication use, low-density lipoprotein cholesterol, and high-density lipoprotein cholesterol). Subgroup analyses were performed by age and sex. Assuming linear association, HRs per 1-unit increase in the TyG index were evaluated. Cochran’s Q statistic was used to identify the presence of interactions in the magnitude of the HR between each subgroup. Restricted cubic spline curves were created with 3 default knots at the 10th, 50th, and 90th percentiles. As an exploratory test, the associations of individual fasting glucose and triglyceride levels with primary cardiovascular events were evaluated, respectively. For this analysis, fasting glucose levels was divided into 4 categories (< 100 [reference], 100–125, 126–139, and **≥** 140 mg/dL) and fasting triglyceride levels into 5 categories (< 50 [reference], 50–99, 100–149, 150–199, 200–499, and ≥ 500 mg/dL**)**. A 2-sided *P* value of < 0.05 was considered to indicate statistical significance. All statistical analyses were performed using SAS version 9.4.

## Results

### Baseline characteristics of the study population

Overall, 155,167 cancer survivors (mean age 59.9 ± 12.0 years, female 59.1%) comprised the study population. Their baseline demographic and biochemical profiles are presented in Table 1. In our cohort, stomach (18.8%), thyroid (15.6%), breast (13.9%), and colorectal cancer (11.7%) were the most common types of cancer. As the TyG index increased, gradual increases in systolic blood pressure, BMI, and total cholesterol were observed, as well as a gradual decrease in high-density lipoprotein cholesterol. Likewise, the high TyG index group exhibited more alcohol consumption, a higher proportion of current smokers, and less physical activity. During a median 10 years (mean 9.6 years) of follow-up, 13,279 CVD hospitalizations occurred.

**Table 1:** Baseline characteristics of the study population by TyG index

Variable		Total n (%)	TyG index					
			< 8	8–8.4	8.5–8.9	9.0–9.4	9.5–9.9	≥ 10
Participants	Number (%)	155,167	26,410 (17.0)	49,933 (32.2)	47,578 (30.7)	22,676 (14.6)	6781 (4.4)	1789 (1.2)
Age, years	Mean ± SD	59.9 ± 12.9	56.1 ± 12.9	59.6 ± 12.1	61.3 ± 11.5	61.6 ± 11.2	61.2 ± 11.1	60.2 ± 11.1
Sex	Men	63,406 (40.9)	9368 (35.5)	19,652 (39.4)	19,690 (41.4)	10,194 (45.0)	3455 (51.0)	1047 (58.5)
	Women	91,761 (59.1)	17,042 (64.5)	30,281 (60.6)	27,888 (58.6)	12,482 (55.0)	3326 (49.0)	742 (41.5)
Systolic BP, mmHg	Mean ± SD	123.6 ± 15.8	118.1 ± 14.9	121.9 ± 15.5	125.2 ± 15.6	127.7 ± 15.6	129.5 ± 15.7	131.4 ± 16.1
Body mass index, kg/m^2^	Mean ± SD	23.3 ± 3.1	22.0 ± 2.8	22.8 ± 3.0	23.7 ± 3.1	24.6 ± 3.0	25.0 ± 3.0	25.1 ± 3.0
Fasting glucose, mg/dL	Mean ± SD	96.8 ± 18.7	88.0 ± 10.3	93.0 ± 11.7	97.8 ± 14.6	104.4 ± 21.3	115.0 ± 32.3	142.8 ± 63.6
TC, mg/dL	Mean ± SD	195.2 ± 37.3	178.7 ± 32.9	190.1 ± 34.2	200.4 ± 36.6	207.5 ± 38.6	211.5 ± 40.1	225.4 ± 47.2
TG, mg/dL	Mean ± SD	124.0 ± 77.0	53.7 ± 11.3	85.4 ± 15.0	130.2 ± 24.1	198.3 ± 40.6	299.1 ± 71.0	471.5 ± 167.6
HDL-C, mg/dL	Mean ± SD	55.4 ± 14.1	61.6 ± 14.7	57.9 ± 13.8	53.7 ± 12.9	49.4 ± 12.2	46.6 ± 11.9	45.6 ± 12.4
LDL-C, mg/dL	Mean ± SD	114.8 ± 34.0	106.0 ± 29.7	114.7 ± 31.6	120.4 ± 34.4	118.4 ± 36.5	105.4 ± 38.8	87.8 ± 45.3
TyG index	Mean ± SD	8.5 ± 0.6	7.7 ± 0.2	8.3 ± 0.1	8.7 ± 0.1	9.2 ± 0.1	9.7 ± 0.1	10.3 ± 0.3
Body mass index, kg/m^2^	< 18.5	7746 (5.0)	2466 (9.3)	3217 (6.4)	1595 (3.4)	369 (1.6)	81 (1.2)	18 (1.0)
	18.5–24.9	104,698 (67.5)	20,444 (77.4)	36,171 (72.4)	30,912 (65.0)	12,880 (56.8)	3419 (50.4)	872 (48.7)
	25-29.9	38,846 (25.0)	3305 (12.5)	9730 (19.5)	13,690 (28.8)	8420 (37.1)	2895 (42.7)	806 (45.1)
	≥ 30	3,877 (2.5)	195 (0.7)	815 (1.6)	1,381 (2.9)	1,007 (4.4)	386 (5.7)	93 (5.2)
Smoking status	Missing	767 (0.5)	140 (0.5)	222 (0.4)	244 (0.5)	114 (0.5)	34 (0.5)	13 (0.7)
	Never smoker	113,557 (73.2)	20,650 (78.2)	37,352 (74.8)	34,510 (72.5)	15,679 (69.1)	4337 (64.0)	1,029 (57.5)
	Past smoker	25,637 (16.5)	3816 (14.4)	8058 (16.1)	8007 (16.8)	4074 (18.0)	1292 (19.1)	390 (21.8)
	Current smoker	15,206 (9.8)	1804 (6.8)	4301 (8.6)	4817 (10.1)	2809 (12.4)	1118 (16.5)	357 (20.0)
Alcohol consumption frequency, times/week	Missing	1,628 (1.0)	329 (1.2)	527 (1.1)	488 (1.0)	220 (1.0)	50 (0.7)	14 (0.8)
	< 1	112,803 (72.7)	19,437 (73.6)	37,115 (74.3)	34,971 (73.5)	15,918 (70.2)	4380 (64.6)	982 (54.9)
	1–2	26,995 (17.4)	4844 (18.3)	8483 (17.0)	7937 (16.7)	4024 (17.7)	1302 (19.2)	405 (22.6)
	3–4	7897 (5.1)	1057 (4.0)	2211 (4.4)	2384 (5.0)	1429 (6.3)	607 (9.0)	209 (11.7)
	≥ 5	5844 (3.8)	743 (2.8)	1597 (3.2)	1798 (3.8)	1085 (4.8)	442 (6.5)	179 (10.0)
Moderate to vigorous physical activity, times/week	None	79,816 (51.4)	12,757 (48.3)	25,292 (50.7)	24,994 (52.5)	12,109 (53.4)	3668 (54.1)	996 (55.7)
	1–2	22,827 (14.7)	4049 (15.3)	7357 (14.7)	6895 (14.5)	3269 (14.4)	1005 (14.8)	252 (14.1)
	≥ 3	52,524 (33.8)	9604 (36.4)	17,284 (34.6)	15,689 (33.0)	7298 (32.2)	2108 (31.1)	541 (30.2)
Income status^*^, quartile	1st quartile (low)	29,404 (18.9)	5142 (19.5)	9506 (19.0)	8916 (18.7)	4187 (18.5)	1291 (19.0)	362 (20.2)
	2nd quartile	24,742 (15.9)	4366 (16.5)	7951 (15.9)	7325 (15.4)	3622 (16.0)	1122 (16.5)	356 (19.9)
	3rd quartile	36,814 (23.7)	6176 (23.4)	11,620 (23.3)	11,487 (24.1)	5480 (24.2)	1613 (23.8)	438 (24.5)
	4th quartile (high)	64,207 (41.4)	10,726 (40.6)	20,856 (41.8)	19,850 (41.7)	9387 (41.4)	2755 (40.6)	633 (35.4)
Use of lipid lowering medication	No	143,222 (92.3)	25,363 (96.0)	46,627 (93.4)	43,341 (91.1)	20,260 (89.3)	6026 (88.9)	1605 (89.7)
	Yes	11,945 (7.7)	1047 (4.0)	3306 (6.6)	4237 (8.9)	2416 (10.7)	755 (11.1)	184 (10.3)

BP: blood pressure; HDL-C: high-density lipoprotein cholesterol; LDL-C: low-density lipoprotein cholesterol; TC: total cholesterol; TG: triglyceride; TyG index: triglyceride-glucose index

^*^Income status was stratified by the quartile of all NHIS beneficiaries (not quartiles of the study population)

### Associations between the TyG index and cardiovascular events

For primary cardiovascular events, a gradual risk elevation was observed across the TyG index levels. Compared to a TyG index < 8, the HRs were 1.08 (95% CI 1.01–1.14) for a TyG index of 8.0–8.4; 1.10 (95% CI 1.03–1.17) for a TyG index of 8.5–8.9; 1.23 (95% CI 1.15–1.31) for a TyG index of 9.0-9.4; 1.34 (95% CI 1.23–1.47) for a TyG index of 9.5–9.9; and 1.55 (95% CI 1.35–1.79) for a TyG index **≥** 10 after controlling for age, sex, behavioral factors, and other laboratory findings (Table 2). Subgroup analyses by age and sex also revealed similar positive associations (Additional file 1: Table S1). As a sensitivity test, we performed the same analyses after excluding those who died in the first 3 years of follow-up and found similar results (Additional file 1: Table S2).

**Table 2:** HRs for primary cardiovascular events by the TyG index

TyG index	No. of events	Crude rate, per 10^5^ person-years	Age- and sex-adjusted		Multivariate-adjusted^a^		Multivariate-adjusted^b^	
			HR (95% CI)	P value	HR (95% CI)	P value	HR (95% CI)	P value
< 8	1511	626	1.00 (Reference)		1.00 (Reference)		1.00 (Reference)	
8.0–8.4	3917	876	1.15 (1.09–1.22)	< 0.001	1.14 (1.08–1.21)	< 0.001	1.08 (1.01–1.14)	0.015
8.5–8.9	4350	1028	1.25 (1.18–1.33)	< 0.001	1.23 (1.16–1.31)	< 0.001	1.10 (1.03–1.17)	0.003
9.0-9.4	2459	1230	1.48 (1.39–1.58)	< 0.001	1.45 (1.36–1.54)	< 0.001	1.23 (1.15–1.31)	< 0.001
9.5–9.9	807	1365	1.65 (1.52–1.80)	< 0.001	1.60 (1.47–1.75)	< 0.001	1.34 (1.23–1.47)	< 0.001
≥ 10	235	1526	1.92 (1.68–2.21)	< 0.001	1.85 (1.61–2.12)	< 0.001	1.55 (1.35–1.79)	< 0.001

^a^Adjustment for age, sex, household income, and behavioral factors (alcohol consumption, smoking status, and physical activity)

^b^Adjustment for age, sex, household income, behavioral factors, and cardiometabolic factors (systolic blood pressure, body mass index, lipid-lowering medication use, low-density lipoprotein cholesterol, and high-density lipoprotein cholesterol)

CVD: cardiovascular disease; HR: hazard ratio; TyG index: triglyceride-glucose index

Primary cardiovascular event refers to hospitalization due to major cardiovascular disease (composite of either ischemic heart disease, stroke, or heart failure)

When we analyzed the associations of the TyG index with subtypes of CVD, graded positive associations between the TyG index and clinical outcomes were particularly evident for atherosclerotic cardiovascular disease (ASCVD), such as IHD (including AMI) and ischemic stroke, but not for hemorrhagic stroke or heart failure (Additional file 1: Table S3). For instance, the adjusted HRs for AMI were 1.23 (95% CI 0.97–1.55) for a TyG index of 8.0–8.4; 1.40 (95% CI 1.11–1.77) for a TyG index of 8.5–8.9; 1.75 (95% CI 1.36–2.23) for a TyG index of 9.0–9.4; 2.07 (95% CI 1.53–2.80) for a TyG index of 9.5–9.9; and 2.58 (95% CI 1.65–4.02) for a TyG index **≥** 10. The adjusted HRs for ischemic stroke were 1.13 (95% CI 1.02–1.25) for a TyG index of 8.0–8.4, 1.15 (95% CI 1.04–1.28) for a TyG index of 8.5–8.9, 1.34 (95% CI 1.20–1.51) for a TyG index of 9.0–9.4, 1.56 (95% CI 1.35–1.82) for a TyG index of 9.5–9.9, and 1.92 (95% CI 1.53–2.41) for a TyG index **≥** 10 compared to the reference group with a TyG index < 8. Conversely for hemorrhagic stroke and heart failure, most of the TyG categories included the null with *P* values > 0.05, indicating a non-significant association.

**Fig. 1:**
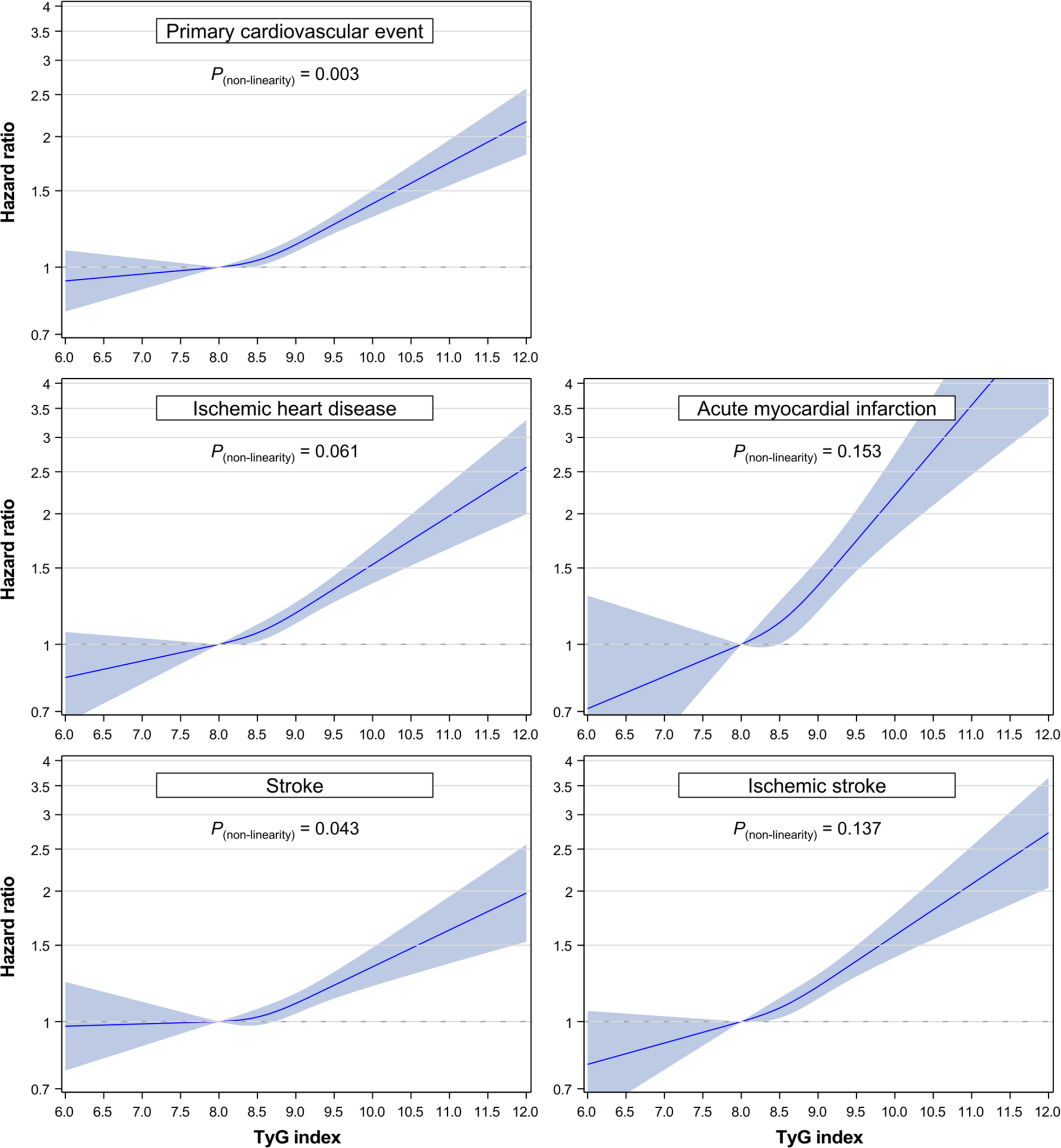
HRs for cardiovascular events using spline analyses. Restricted cubic splines of the TyG index with 3 knots (10th, 50th, and 90th percentiles) and 8.0 as a reference were used. HRs and 95% CIs were calculated using Cox proportional hazards models after adjustment for sex, age at baseline, smoking status, alcohol consumption frequency, physical activity, household income, systolic blood pressure, body mass index, lipid-lowering medication use, low-density lipoprotein cholesterol, and high-density lipoprotein cholesterol. HR: hazard ratio; TyG index: triglyceride-glucose index

A restricted cubic spline curve for primary cardiovascular events showed a similar graded positive association to that observed in the categorical analysis (Fig. 1). In particular, the slope of association was steeper and the linear association was more apparent for ASCVD subtypes including AMI and ischemic stroke (*P* value for non-linearity > 0.05) than for non-ASCVD subtypes (Fig. 1 and Additional file 1: Fig. S2).

**Fig. 2:**
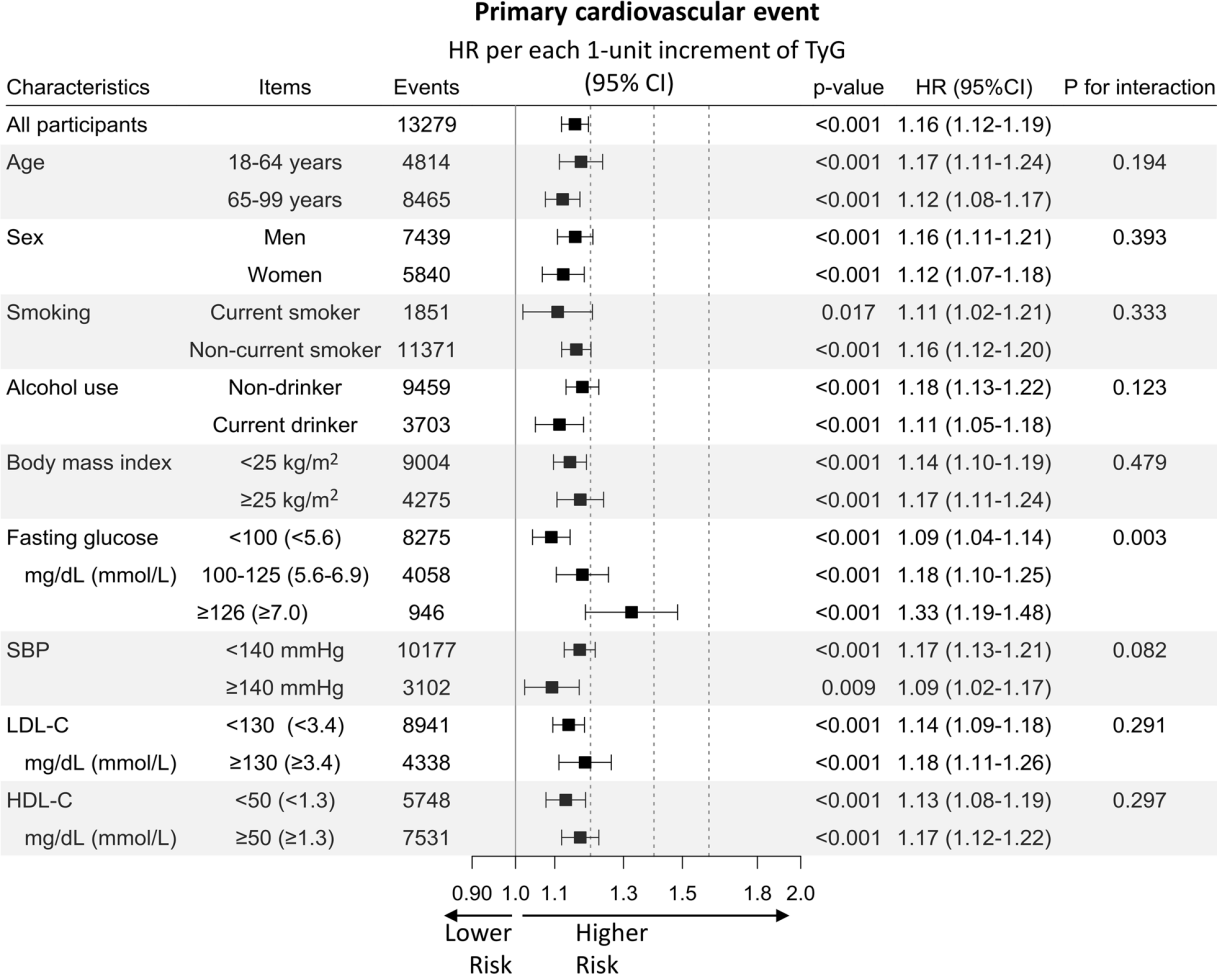
HRs per 1-unit increase in the TyG index for primary cardiovascular events by confounders. HRs and 95% CIs were calculated using Cox proportional hazards models after adjustment for the same variables as in Fig. 1. HR: hazard ratio; TyG index: triglyceride-glucose index

**Fig. 3:**
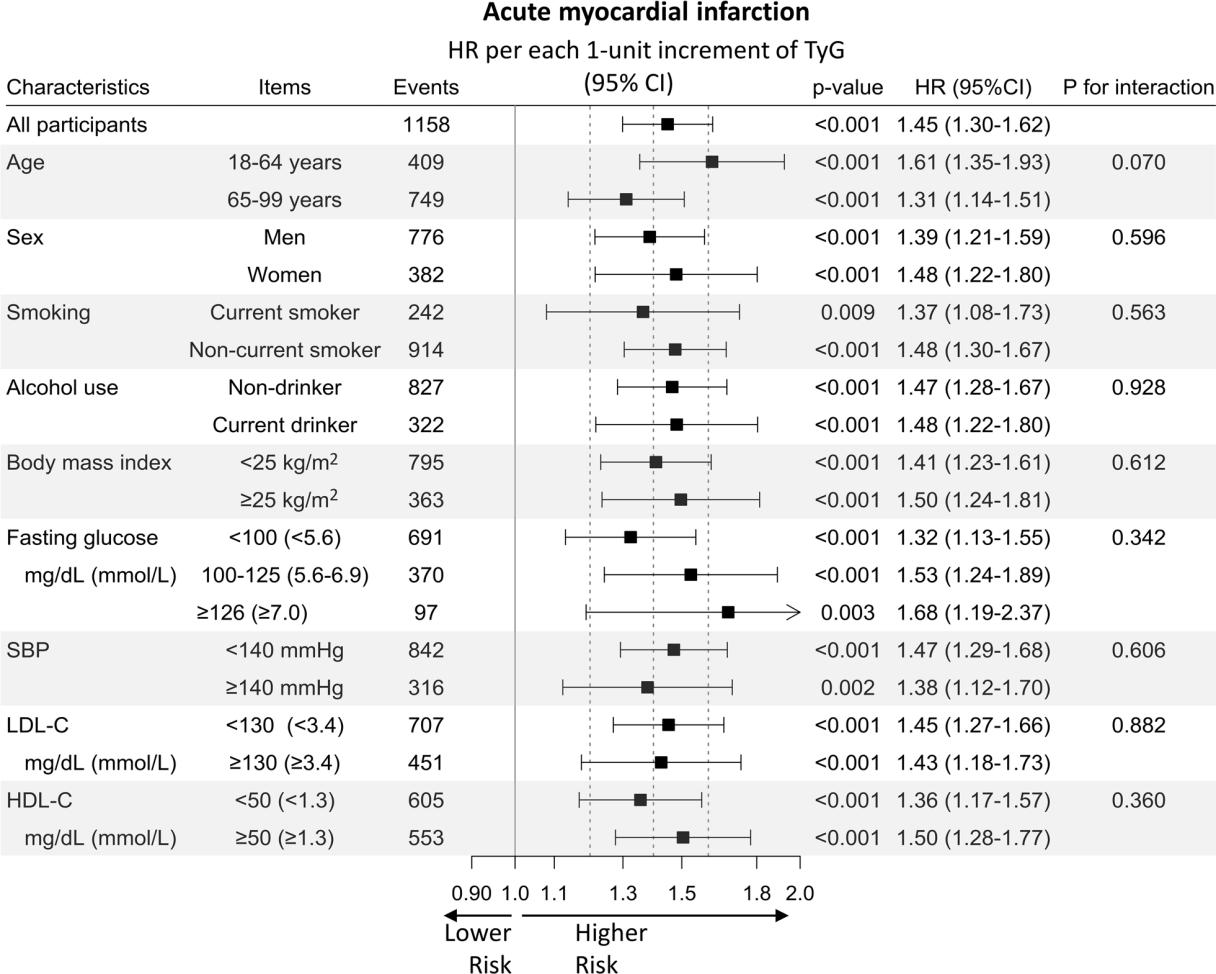
HRs per 1-unit increase in the TyG index for acute myocardial infarction by confounders. HRs and 95% CIs were calculated using Cox proportional hazards models after adjustment for the same variables as in Fig. 1. HR: hazard ratio; TyG index: triglyceride-glucose index

When we assumed a linear association, each 1-unit increase in the TyG index was associated with a 16% increase in primary cardiovascular events (HR 1.16; 95% CI 1.12–1.19). The association was the strongest for AMI. Each 1-unit increase in the TyG index increased the risk of hospitalization for AMI by 45% (HR, 1.45; 95% CI 1.30–1.62), for ischemic stroke by 23% (HR 1.23; 95% CI 1.17–1.30), for IHD by 20% (HR 1.20; 95% CI 1.14–1.26), and for overall stroke by 13% (HR 1.13, 95% CI 1.08–1.19), after adjustment for baseline covariates. The associations between TyG index and primary cardiovascular events did not differ between subgroups (*P*_interaction_ > 0.05), except for glycemic status, wherein a stronger association was found in higher glycemic group (*P*_interaction_=0.003) (Figs. 2 and 3).

### Exploratory analyses: associations of individual fasting glucose and triglyceride levels with primary cardiovascular events

Regarding fasting glucose, risk elevation was only evident for fasting glucose levels **≥** 140 mg/dL after adjustment for baseline covariates compared to the reference group with fasting glucose levels < 100 mg/dL (Additional file 1: Table S4). For triglyceride levels, a potentially gradual risk elevation was demonstrated, and triglyceride levels **≥** 500 mg/dL were associated with a 59% higher risk of primary cardiovascular events (HR 1.59, 95% CI 1.26–2.01) compared to the group with triglyceride levels < 50 mg/dL (Additional file 1: Table S4). When 6 combined fasting glucose-triglyceride categories were analyzed, the group with fasting glucose ≥ 140 mg/dL and TG < 100 mg/dL was not associated with higher risk, while the group with fasting glucose ≥ 140 mg/dL and TG ≥ 500 mg/dL was associated with a 2.63-fold higher risk (Additional file 1: Table S5).

## Discussion

The current study demonstrated that the TyG index was positively associated with cardiovascular events in a dose-dependent manner in a large nationwide cohort involving over 150,000 cancer survivors. Similar findings were observed across different age and sex subgroups. Notably, graded positive associations were evident for ASCVD subtypes, such as IHD, AMI, and ischemic stroke, but not for hemorrhagic stroke or heart failure. Furthermore, the slope of the association was steeper for AMI and ischemic stroke. To our knowledge, the current study is the first to demonstrate the risk of CVD occurrence based on the baseline TyG index among cancer survivors.

IHD, especially AMI, had a strong positive association with the TyG index in cancer survivors. Several studies have prospectively examined the association in non-CVD patients [22, 23] and in the general population [14, 24–27]. In studies of the general population, the TyG index was generally positively associated with IHD events, although the effect size varied across studies. A study that showed the strongest potential association (HR for the highest quartile = 2.28, compared to the lowest quartile) did not adjust for lipid levels [26]. A US study in male non-CVD patients found that the association of higher IHD mortality with a high TyG index disappeared after further adjustment of non-HDL-cholesterol [23]. In the current study, the associations were strongly maintained after adjustment for lipid levels. It is difficult to compare the effect size of the current study with those of previous studies due to differences in the outcome definition and, especially, categorizations of the TyG index. A large-scale study with 5,593,134 individuals from the Korean general population reported an HR of myocardial infarction (ICD-10: I21-I22) for the highest quartile of 1.31, compared to the lowest quartile, while, in the current study, the HRs associated with a TyG index ≥ 10 were 1.67 for IHD (ICD-10: I20-I25), and 2.58 for AMI (ICD-10: I21). Overall, the pattern of association—namely, a positive association—was similar, but whether there are differences in the effect size between general population and cancer patients requires further study to confirm.

Ischemic stroke showed a positive association with the TyG index in cancer survivors. Compared to IHD, fewer prospective studies have examined the associations in CVD patients [28] and in the general population [27, 29, 30]; in those studies, the TyG index was independently associated with a higher risk of ischemic stroke, in accordance with the current study. Among studies reporting both myocardial infarction and ischemic stroke simultaneously [27, 28], the potential associations were stronger for myocardial infarction than for ischemic stroke, in line with the current study. Regarding the strength of associations, there seemed to be some differences among studies, including ours [27–30]; the Rural Chinese Cohort Study reported an HR of 1.95 for the highest quartile of the TyG index compared to the lowest quartile [30], while the corresponding HR was 1.30 in the Kailuan study [27]. For hemorrhagic stroke, limited studies exist. Two research groups independently reported no associations for intracerebral hemorrhage in the same Chinese general population [27, 29], which was reaffirmed in our study. Hemorrhagic stroke (intracerebral hemorrhage or subarachnoid hemorrhage) is known to be affected by hemodynamic factors, rather than atherosclerotic factors. In this regard, it is considered that the TyG index, an index for insulin resistance, does not sufficiently reflect the pathophysiology of hemorrhagic stroke. Considering differences in the association between ischemic and hemorrhagic strokes, the association between TyG index and overall stroke may vary across ethnic and regional groups with different distribution of stroke subtypes.

As for heart failure, no prior studies have tested the association between the TyG index and heart failure; furthermore, inconsistent associations between insulin resistance and heart failure have been reported [31–33]. In the current study, no significant association was demonstrated with regard to heart failure. One plausible explanation is that heart failure is a complex clinical syndrome that originates from different etiologies.

In cancer survivors, the TyG index had clear positive-graded associations with ASCVD, such as IHD and ischemic stroke events. During recent decades, cancer outcomes have substantially improved [34, 35], and an increasing number of cancer survivors are experiencing morbidity and mortality originating from CVD [1–3, 36]. However, cancer patients have traditionally been excluded from most clinical trials for lipid lowering, anti-diabetes, or anti-hypertensive agents. Furthermore, metabolic parameters, such as lipid profile or glycemic status, have still not been adequately addressed and tend to be undertreated in cancer survivors. One study revealed that over half (61.7%) of lung cancer survivors for whom statin therapy was indicated were not on treatment [37]. Moreover, counseling about a healthy lifestyle (diet, exercise, or smoking) was infrequently performed among cancer survivors compared to those without cancer [38]. In this context, a well-designed large cohort study might be helpful for the understanding of this important but under-addressed health issue. Furthermore, our results imply that cancer survivors warrant proactive surveillance and management for CVD, just like the general population.

In the current study, we categorized the TyG index based on absolute values (not relative values such as quartiles) anticipating that our study data could be used as a reference in practice. Most previous studies categorized the TyG index level as tertiles or quartiles [10–14, 25–29, 39]. Although such a method can guarantee the same sample size across the groups, the cut-off values might be different across study populations, limiting their use in practice.

The precise mechanism underlying the association between an elevated TyG index (insulin resistance) and CVD events is not clear. However, several possible explanations have been suggested. First, insulin resistance is linked to endothelial dysfunction. Insulin exerts vascular actions (stimulation of the production of nitric oxide by the endothelium) beyond its metabolic actions. Thus, nitric oxide deficiency in insulin resistance could lead to insufficient vasodilation, inflammation, glucotoxicity, and lipotoxicity, resulting in a vicious cycle [40, 41]. Second, insulin resistance could stimulate hyperplasia and hypertrophy of smooth muscle cells in arterial walls, which could play a role in the development of CVD. High levels of insulin function as a potent growth factor that affects the MAPK pathway, leading to stimulation of vascular smooth muscle cell growth and activating inflammatory pathways [41, 42]. Third, insulin resistance is associated with atherogenesis (plaque progression and rupture) [10, 11, 39]. Finally, insulin resistance is associated with a cluster of metabolic abnormalities (visceral obesity, hypertension, dyslipidemia, and nonalcoholic fatty liver disease). Each component could independently contribute to the development of CVD [40].

Several limitations of the current study need to be discussed. First, the observational study design itself limits causal inferences. However, in the absence of randomized controlled trial data for metabolic profiles among cancer survivors, well-designed large population-based real-world data could play an important role. Second, we did not have data for insulin; thus, the TyG index could not be directly compared with the hyperinsulinemic-euglycemic clamp test or HOMA-IR. Although the hyperinsulinemic-euglycemic clamp test is the gold-standard method for evaluating insulin resistance, it entails additional sampling and costs, limiting its wide application at the population level. Conversely, the TyG index is a feasible and cost-effective index that uses already sampled fasting glucose and triglyceride levels. Moreover, it has reliable sensitivity and specificity for identifying insulin resistance [7, 43]. Several previous studies also have shown that the TyG index could better predict metabolic syndrome or subclinical atherosclerosis than HOMA-IR [8, 12, 39, 44]. Lastly, we did not evaluate whether the associations differed by cancer subtypes in the current study. Subsequent studies demonstrating the relationship between the TyG index and CVD in each cancer subtype would be valuable to solidify these results.

## Conclusions

In summary, an elevated TyG index was associated with CVD events (particularly ASCVD) in a dose-dependent manner in a large nationwide cohort of 0.15 million cancer survivors. The TyG index could serve as a simple surrogate marker for the risk stratification of future CVD events in cancer survivors.

## Supplementary Information

**Additional file 1.** Additional Figures and Tables.

## Data Availability

The data supporting the findings of this study are available from the NHIS for the researchers, when their study proposal is reviewed and approved by the NHIS.

## Abbreviations

CVD: Cardiovascular disease
TyG: Triglyceride-glucose
NHIS: National Health Insurance Service
ICD: International Classification of Disease
BMI: Body mass index
IHD: Ischemic heart disease
AMI: Acute myocardial infarction
HR: Hazard ratio
ASCVD: Atherosclerotic cardiovascular disease
