# Supplementary material for: Associations between the triglyceride-glucose index and cardiovascular disease in over 150,000 cancer survivors: a population-based cohort study
Source: Cardiovasc Diabetol. 2022 Apr 16;21:52. doi: 10.1186/s12933-022-01490-z (PMC9013459; doi:10.1186/s12933-022-01490-z)
Supplement: Supplementary file 1 — Additional file 1. Additional Figures and Tables. [file 12933_2022_1490_MOESM1_ESM.docx]

**Figure S1. Flow chart of the study population**

**Figure S2. HRs for cardiovascular events using spline analyses**

Restricted cubic splines of the TyG index with 3 knots (10th, 50th, 90th percentiles) and 8.0 as a reference were used. HRs and 95% confidence intervals were calculated using Cox proportional hazards models after adjustment for sex, age at baseline, smoking status, alcohol consumption frequency, physical activity, household income, systolic blood pressure, body mass index, lipid-lowering medication use, low-density lipoprotein cholesterol, and high-density lipoprotein cholesterol. HR, hazard ratio; TyG index, triglyceride-glucose index.

**Table S1. HRs for primary cardiovascular events by TyG index: subgroup analyses by age and sex**

| **TyG index** | **No. of events** | **Crude rate,**  **per 10^5^ person-year** | **Age- and sex-adjusted** | | **Multivariate-adjusted**^a^ | | **Multivariate-adjusted**^b^ | |
| --- | --- | --- | --- | --- | --- | --- | --- | --- |
|  |  |  | **HR (95% CI)** | **P-value** | **HR (95% CI)** | **P-value** | **HR (95% CI)** | **P-value** |
| **Age strata** |  |  |  |  |  |  |  |  |
| **Aged < 65 years** |  |  |  |  |  |  |  |  |
| < 8 | 599 | 322 | 1.00 (Reference) |  | 1.00 (Reference) |  | 1.00 (Reference) | - |
| 8.0-8.4 | 1,360 | 445 | 1.20 (1.09-1.32) | <0.001 | 1.18 (1.07-1.30) | <0.001 | 1.09 (0.99-1.20) | 0.094 |
| 8.5-8.9 | 1,527 | 564 | 1.37 (1.25-1.51) | <0.001 | 1.35 (1.22-1.48) | <0.001 | 1.14 (1.03-1.26) | 0.009 |
| 9.0-9.4 | 880 | 694 | 1.59 (1.43-1.76) | <0.001 | 1.54 (1.39-1.71) | <0.001 | 1.22 (1.09-1.36) | <0.001 |
| 9.5-9.9 | 346 | 901 | 1.98 (1.73-2.26) | <0.001 | 1.90 (1.66-2.17) | <0.001 | 1.46 (1.27-1.68) | <0.001 |
| ≥10 | 102 | 978 | 2.12 (1.71-2.61) | <0.001 | 1.99 (1.61-2.46) | <0.001 | 1.53 (1.23-1.90) | <0.001 |
| **Aged ≥65 years** |  |  |  |  |  |  |  |  |
| < 8 | 912 | 1,654 | 1.00 (Reference) |  | 1.00 (Reference) |  | 1.00 (Reference) |  |
| 8.0-8.4 | 2,557 | 1,807 | 1.10 (1.02-1.19) | 0.010 | 1.10 (1.02-1.18) | 0.016 | 1.05 (0.98-1.14) | 0.177 |
| 8.5-8.9 | 2,823 | 1,856 | 1.15 (1.07-1.24) | <0.001 | 1.14 (1.06-1.23) | <0.001 | 1.05 (0.97-1.13) | 0.213 |
| 9.0-9.4 | 1,579 | 2,161 | 1.36 (1.25-1.48) | <0.001 | 1.34 (1.24-1.46) | <0.001 | 1.19 (1.10-1.30) | <0.001 |
| 9.5-9.9 | 461 | 2,227 | 1.40 (1.26-1.57) | <0.001 | 1.38 (1.23-1.54) | <0.001 | 1.22 (1.09-1.37) | <0.001 |
| ≥10 | 133 | 2,676 | 1.71 (1.43-2.06) | <0.001 | 1.68 (1.40-2.02) | <0.001 | 1.49 (1.24-1.79) | <0.001 |
| **Sex** |  |  |  |  |  |  |  |  |
| **Men** |  |  |  |  |  |  |  |  |
| < 8 | 889 | 1,138 | 1.00 (Reference) |  | 1.00 (Reference) |  | 1.00 (Reference) |  |
| 8.0-8.4 | 2,174 | 1,334 | 1.11 (1.03-1.21) | 0.006 | 1.11 (1.02-1.19) | 0.012 | 1.04 (0.96-1.12) | 0.379 |
| 8.5-8.9 | 2,413 | 1,468 | 1.26 (1.16-1.36) | <0.001 | 1.24 (1.15-1.34) | <0.001 | 1.09 (1.01-1.18) | 0.032 |
| 9.0-9.4 | 1,340 | 1,563 | 1.44 (1.32-1.57) | <0.001 | 1.41 (1.30-1.54) | <0.001 | 1.19 (1.09-1.30) | <0.001 |
| 9.5-9.9 | 467 | 1,603 | 1.58 (1.41-1.77) | <0.001 | 1.53 (1.37-1.71) | <0.001 | 1.30 (1.15-1.46) | <0.001 |
| ≥10 | 156 | 1,783 | 1.89 (1.59-2.24) | <0.001 | 1.82 (1.53-2.16) | <0.001 | 1.58 (1.33-1.88) | <0.001 |
| **Women** |  |  |  |  |  |  |  |  |
| < 8 | 622 | 381 | 1.00 (Reference) |  | 1.00 (Reference) |  | 1.00 (Reference) |  |
| 8.0-8.4 | 1,743 | 613 | 1.17 (1.07-1.29) | <0.001 | 1.17 (1.06-1.28) | 0.001 | 1.11 (1.01-1.22) | 0.024 |
| 8.5-8.9 | 1,937 | 749 | 1.19 (1.09-1.30) | <0.001 | 1.18 (1.08-1.29) | <0.001 | 1.07 (0.97-1.18) | 0.153 |
| 9.0-9.4 | 1,119 | 980 | 1.43 (1.30-1.58) | <0.001 | 1.41 (1.28-1.56) | <0.001 | 1.22 (1.10-1.35) | <0.001 |
| 9.5-9.9 | 340 | 1,135 | 1.63 (1.42-1.86) | <0.001 | 1.59 (1.40-1.82) | <0.001 | 1.34 (1.16-1.53) | <0.001 |
| ≥10 | 79 | 1,187 | 1.77 (1.40-2.24) | <0.001 | 1.73 (1.37-2.18) | <0.001 | 1.42 (1.12-1.80) | 0.004 |

^a^Adjusted for age, sex, household income, and behavioral factors (alcohol consumption, smoking habit, and physical activity)

^b^Adjusted for age, sex, household income, behavioral factors, and cardiometabolic factors (systolic blood pressure, body mass index, lipid-lowering medication use, low-density lipoprotein cholesterol, and high-density lipoprotein cholesterol).

CI, confidence interval; CVD, cardiovascular disease; HR, hazard ratio; TyG index, triglyceride-glucose index.

**Table S2. HRs for primary cardiovascular events after excluding the first 3 years of follow-up (sensitivity analysis)**

| **TyG index** | **Multivariate-adjusted**^a^ | |
| --- | --- | --- |
|  | **HR (95% CI)** | **P-value** |
| **Overall population** |  |  |
| < 8 | 1.00 (Reference) | - |
| 8.0-8.4 | 1.07 (1.01-1.14) | 0.024 |
| 8.5-8.9 | 1.09 (1.02-1.16) | 0.006 |
| 9.0-9.4 | 1.21 (1.13-1.30) | <0.001 |
| 9.5-9.9 | 1.35 (1.23-1.48) | <0.001 |
| ≥10 | 1.52 (1.31-1.75) | <0.001 |
| **Aged < 65 years** |  |  |
| < 8 | 1.00 (Reference) | - |
| 8.0-8.4 | 1.08 (0.98-1.19) | 0.119 |
| 8.5-8.9 | 1.13 (1.02-1.25) | 0.015 |
| 9.0-9.4 | 1.22 (1.09-1.36) | <0.001 |
| 9.5-9.9 | 1.46 (1.27-1.68) | <0.001 |
| ≥10 | 1.51 (1.21-1.88) | <0.001 |
| **Aged ≥ 65 years** |  |  |
| < 8 | 1.00 (Reference) | - |
| 8.0-8.4 | 1.05 (0.97-1.14) | 0.223 |
| 8.5-8.9 | 1.04 (0.96-1.13) | 0.302 |
| 9.0-9.4 | 1.17 (1.07-1.28) | 0.001 |
| 9.5-9.9 | 1.23 (1.09-1.38) | 0.001 |
| ≥10 | 1.44 (1.19-1.75) | <0.001 |
| **Men** |  |  |
| < 8 | 1.00 (Reference) | - |
| 8.0-8.4 | 1.03 (0.95-1.12) | 0.415 |
| 8.5-8.9 | 1.09 (1.00-1.18) | 0.045 |
| 9.0-9.4 | 1.17 (1.07-1.29) | 0.001 |
| 9.5-9.9 | 1.30 (1.16-1.47) | <0.001 |
| ≥10 | 1.55 (1.30-1.85) | <0.001 |
| **Women** |  |  |
| < 8 | 1.00 (Reference) | - |
| 8.0-8.4 | 1.10 (1.01-1.21) | 0.039 |
| 8.5-8.9 | 1.06 (0.96-1.17) | 0.231 |
| 9.0-9.4 | 1.20 (1.08-1.34) | 0.001 |
| 9.5-9.9 | 1.34 (1.16-1.54) | <0.001 |
| ≥10 | 1.37 (1.07-1.75) | 0.013 |

^a^Adjusted for age, sex, household income, behavioral factors, and cardiometabolic factors (systolic blood pressure, body mass index, lipid-lowering medication use, low-density lipoprotein cholesterol, and high-density lipoprotein cholesterol).

**Table S3. HRs for CVD subtypes by the TyG index**

| **TyG index** | **No. of events** | **Crude rate,**  **per 10^5^ person-year** | **Age- and sex-adjusted** | | **Multivariate-adjusted**^a^ | | **Multivariate-adjusted**^b^ | |
| --- | --- | --- | --- | --- | --- | --- | --- | --- |
|  |  |  | **HR (95% CI)** | **P-value** | **HR (95% CI)** | **P-value** | **HR (95% CI)** | **P-value** |
| **IHD (I20-I25)** | | | | | | | | |
| < 8 | 668 | 274 | 1.00 (Reference) |  | 1.00 (Reference) |  | 1.00 (Reference) |  |
| 8.0-8.4 | 1744 | 384 | 1.20 (1.10-1.31) | <0.001 | 1.19 (1.09-1.30) | <0.001 | 1.09 (0.99-1.19) | 0.075 |
| 8.5-8.9 | 2062 | 479 | 1.41 (1.29-1.53) | <0.001 | 1.39 (1.27-1.51) | <0.001 | 1.16 (1.06-1.26) | 0.002 |
| 9.0-9.4 | 1179 | 577 | 1.65 (1.50-1.81) | <0.001 | 1.63 (1.48-1.79) | <0.001 | 1.27 (1.15-1.40) | <0.001 |
| 9.5-9.9 | 404 | 667 | 1.88 (1.66-2.13) | <0.001 | 1.85 (1.64-2.10) | <0.001 | 1.44 (1.27-1.64) | <0.001 |
| ≥10 | 118 | 746 | 2.10 (1.73-2.55) | <0.001 | 2.07 (1.70-2.52) | <0.001 | 1.67 (1.37-2.04) | <0.001 |
| **AMI (I21)** | | | | | | | | |
| < 8 | 94 | 38 | 1.00 (Reference) |  | 1.00 (Reference) |  | 1.00 (Reference) |  |
| 8.0-8.4 | 301 | 65 | 1.43 (1.13-1.80) | 0.002 | 1.40 (1.11-1.76) | 0.005 | 1.23 (0.97-1.55) | 0.088 |
| 8.5-8.9 | 395 | 90 | 1.85 (1.48-2.32) | <0.001 | 1.80 (1.43-2.25) | <0.001 | 1.40 (1.11-1.77) | 0.004 |
| 9.0-9.4 | 255 | 122 | 2.46 (1.94-3.12) | <0.001 | 2.39 (1.88-3.03) | <0.001 | 1.75 (1.36-2.23) | <0.001 |
| 9.5-9.9 | 87 | 140 | 2.80 (2.09-3.75) | <0.001 | 2.72 (2.03-3.65) | <0.001 | 2.07 (1.53-2.80) | <0.001 |
| ≥10 | 26 | 160 | 3.22 (2.08-4.97) | <0.001 | 3.16 (2.05-4.89) | <0.001 | 2.58 (1.65-4.02) | <0.001 |
| **Total stroke (I60-I69)** | | | | | | | | |
| < 8 | 726 | 297 | 1.00 (Reference) |  | 1.00 (Reference) |  | 1.00 (Reference) |  |
| 8.0-8.4 | 1918 | 421 | 1.14 (1.04-1.24) | 0.003 | 1.12 (1.03-1.23) | 0.007 | 1.09 (1.00-1.18) | 0.059 |
| 8.5-8.9 | 2008 | 463 | 1.15 (1.06-1.25) | 0.001 | 1.13 (1.04-1.23) | 0.005 | 1.06 (0.97-1.15) | 0.230 |
| 9.0-9.4 | 1132 | 550 | 1.35 (1.23-1.48) | <0.001 | 1.32 (1.20-1.44) | <0.001 | 1.19 (1.08-1.32) | <0.001 |
| 9.5-9.9 | 383 | 628 | 1.58 (1.39-1.79) | <0.001 | 1.52 (1.34-1.72) | <0.001 | 1.36 (1.20-1.55) | <0.001 |
| ≥10 | 107 | 673 | 1.79 (1.46-2.19) | <0.001 | 1.70 (1.39-2.08) | <0.001 | 1.50 (1.22-1.84) | <0.001 |
| **Ischemic stroke (I63)** | | | | | | | | |
| < 8 | 218 | 197 | 1.00 (Reference) |  | 1.00 (Reference) |  | 1.00 (Reference) |  |
| 8.0-8.4 | 481 | 297 | 1.20 (1.08-1.33) | <0.001 | 1.18 (1.07-1.31) | 0.002 | 1.13 (1.02-1.25) | 0.023 |
| 8.5-8.9 | 417 | 347 | 1.28 (1.16-1.42) | <0.001 | 1.26 (1.14-1.39) | <0.001 | 1.15 (1.04-1.28) | 0.009 |
| 9.0-9.4 | 213 | 425 | 1.55 (1.39-1.74) | <0.001 | 1.51 (1.35-1.69) | <0.001 | 1.34 (1.20-1.51) | <0.001 |
| 9.5-9.9 | 68 | 488 | 1.83 (1.58-2.11) | <0.001 | 1.76 (1.52-2.03) | <0.001 | 1.56 (1.35-1.82) | <0.001 |
| ≥10 | 18 | 570 | 2.26 (1.81-2.83) | <0.001 | 2.16 (1.72-2.70) | <0.001 | 1.92 (1.53-2.41) | <0.001 |
| **Hemorrhagic stroke (I60-I62)** | | | | | | | | |
| < 8 | 484 | 88 | 1.00 (Reference) |  | 1.00 (Reference) |  | 1.00 (Reference) |  |
| 8.0-8.4 | 1361 | 104 | 0.98 (0.84-1.15) | 0.821 | 0.97 (0.83-1.14) | 0.751 | 0.97 (0.83-1.14) | 0.737 |
| 8.5-8.9 | 1510 | 95 | 0.83 (0.70-0.98) | 0.026 | 0.82 (0.69-0.96) | 0.016 | 0.81 (0.68-0.96) | 0.013 |
| 9.0-9.4 | 878 | 102 | 0.87 (0.72-1.06) | 0.164 | 0.85 (0.71-1.03) | 0.101 | 0.81 (0.67-0.99) | 0.042 |
| 9.5-9.9 | 299 | 109 | 0.96 (0.73-1.26) | 0.768 | 0.92 (0.70-1.21) | 0.562 | 0.83 (0.62-1.10) | 0.185 |
| ≥10 | 91 | 111 | 1.02 (0.63-1.65) | 0.931 | 0.97 (0.60-1.56) | 0.886 | 0.79 (0.49-1.29) | 0.350 |
| **Heart failure (I11, I13, I255, I42, I50)** | | | | | | | | |
| < 8 | 235 | 95 | 1.00 (Reference) |  | 1.00 (Reference) |  | 1.00 (Reference) |  |
| 8.0-8.4 | 560 | 121 | 0.98 (0.84-1.15) | 0.825 | 0.97 (0.83-1.13) | 0.714 | 0.95 (0.81-1.11) | 0.518 |
| 8.5-8.9 | 608 | 139 | 1.01 (0.87-1.17) | 0.902 | 0.99 (0.85-1.15) | 0.894 | 0.94 (0.80-1.10) | 0.419 |
| 9.0-9.4 | 350 | 168 | 1.21 (1.02-1.42) | 0.027 | 1.18 (1.00-1.39) | 0.057 | 1.06 (0.89-1.26) | 0.543 |
| 9.5-9.9 | 97 | 156 | 1.17 (0.93-1.49) | 0.183 | 1.14 (0.90-1.44) | 0.295 | 0.95 (0.75-1.22) | 0.706 |
| ≥10 | 26 | 160 | 1.35 (0.90-2.02) | 0.149 | 1.28 (0.85-1.92) | 0.238 | 1.01 (0.67-1.52) | 0.979 |

^a^Adjusted for age, sex, household income, and behavioral factors (alcohol consumption, smoking habit, and physical activity)

^b^Adjusted for age, sex, household income, behavioral factors, and cardiometabolic factors (systolic blood pressure, body mass index, lipid-lowering medication use, low-density lipoprotein cholesterol, and high-density lipoprotein cholesterol).

AMI, acute myocardial infarction; CI, confidence interval; CVD, cardiovascular disease; HR, hazard ratio; IHD, ischemic heart disease; TyG index, triglyceride-glucose index.

**Table S4. HRs for primary cardiovascular events by individual fasting glucose and triglyceride levels**

|  | **No. of events** | **Crude rate,**  **per 10^5^ person-years** | **Age- and sex-adjusted** | | **Multivariate-adjusted**^a^ | | **Multivariate-adjusted**^b^ | |
| --- | --- | --- | --- | --- | --- | --- | --- | --- |
|  |  |  | **HR (95% CI)** | ***P* value** | **HR (95% CI)** | ***P* value** | **HR (95% CI)** | ***P* value** |
| **FG, mg/dL** |  |  |  |  |  |  |  |  |
| < 100 | 8,275 | 814 | 1.00 (Reference) |  | 1.00 (Reference) |  | 1.00 (Reference) |  |
| 100-125 | 4,058 | 1,010 | 1.03 (0.99-1.07) | 0.179 | 1.03 (0.99-1.07) | 0.100 | 0.99 (0.95-1.03) | 0.633 |
| 126-139 | 395 | 1,257 | 1.13 (1.02-1.25) | 0.020 | 1.13 (1.02-1.25) | 0.018 | 1.06 (0.96-1.18) | 0.248 |
| ≥140 | 551 | 1,604 | 1.50 (1.38-1.64) | <0.001 | 1.49 (1.36-1.62) | <0.001 | 1.40 (1.28-1.53) | <0.001 |
| **TG, mg/L** |  |  |  |  |  |  |  |  |
| <50 | 491 | 559 | 1.00 (Reference) |  | 1.00 (Reference) |  | 1.00 (Reference) |  |
| 50-99 | 4,643 | 775 | 1.13 (1.03-1.25) | 0.008 | 1.12 (1.02-1.23) | 0.019 | 1.05 (0.96-1.16) | 0.278 |
| 100-149 | 4,074 | 948 | 1.25 (1.14-1.37) | <0.001 | 1.22 (1.11-1.34) | <0.001 | 1.08 (0.99-1.19) | 0.099 |
| 150-199 | 2,091 | 1,076 | 1.41 (1.28-1.56) | <0.001 | 1.37 (1.24-1.51) | <0.001 | 1.17 (1.06-1.29) | 0.002 |
| 200-499 | 1,896 | 1,134 | 1.49 (1.34-1.64) | <0.001 | 1.43 (1.30-1.58) | <0.001 | 1.20 (1.09-1.33) | <0.001 |
| ≥500 | 84 | 1,308 | 1.96 (1.55-2.47) | <0.001 | 1.86 (1.47-2.34) | <0.001 | 1.57 (1.24-1.98) | <0.001 |

^a^Adjusted for age, sex, household income, and behavioral factors (alcohol consumption, smoking status, and physical activity)

^b^Adjusted for age, sex, household income, behavioral factors, and cardiometabolic factors (systolic blood pressure, body mass index, lipid-lowering medication use, low-density lipoprotein cholesterol, high-density lipoprotein cholesterol, TG, and FG, respectively).

CVD, cardiovascular disease; FG, fasting glucose; HR, hazard ratio; TG, triglyceride; TyG index, triglyceride-glucose index.

**Table S5. HRs for primary cardiovascular events by combined fasting glucose and triglyceride levels**

| **FG, mg/dL** | **TG, mg/dL** | **Age- and sex-adjusted** | | **Multivariate-adjusted**^a^ | | **Multivariate-adjusted**^b^ | |
| --- | --- | --- | --- | --- | --- | --- | --- |
|  |  | **HR (95% CI)** | ***P* value** | **HR (95% CI)** | ***P* value** | **HR (95% CI)** | ***P* value** |
| <140 | <100 | 1.00 (Reference) |  | 1.00 (Reference) |  | 1.00 (Reference) |  |
| <140 | 100-499 | 1.19 (1.14-1.23) | <0.001 | 1.17 (1.13-1.21) | <0.001 | 1.06 (1.02-1.10) | 0.001 |
| <140 | ≥500 | 1.68 (1.32-2.13) | <0.001 | 1.61 (1.27-2.05) | <0.001 | 1.40 (1.10-1.78) | 0.007 |
| ≥140 | <100 | 1.12 (0.92-1.37) | 0.246 | 1.11 (0.91-1.36) | 0.284 | 1.05 (0.86-1.28) | 0.620 |
| ≥140 | 100-499 | 1.93 (1.75-2.13) | <0.001 | 1.88 (1.70-2.07) | <0.001 | 1.62 (1.47-1.79) | <0.001 |
| ≥140 | ≥500 | 3.26 (2.00-5.33) | <0.001 | 3.04 (1.86-4.97) | <0.001 | 2.63 (1.61-4.29) | <0.001 |

^a^Adjusted for age, sex, household income, and behavioral factors (alcohol consumption, smoking status, and physical activity)

^b^Adjusted for age, sex, household income, behavioral factors, and cardiometabolic factors (systolic blood pressure, body mass index, lipid-lowering medication use, low-density lipoprotein cholesterol, high-density lipoprotein cholesterol, TG, and FG, respectively).

CVD, cardiovascular disease; FG, fasting glucose; HR, hazard ratio; TG, triglyceride; TyG index, triglyceride-glucose index.
